# Early treatment with nootkatone prevents pressure overload-induced ventricular remodeling and heart failure

**DOI:** 10.3389/fphar.2025.1702627

**Published:** 2026-01-28

**Authors:** Zhongyuan Liu, Zixin Zhou, Wenjing Yuan, Jiajin Li, Jie Tian, Mi Li, Junjun Quan

**Affiliations:** 1 Department of Cardiology, Children’s Hospital of Chongqing Medical University, Chongqing, China; 2 Department of Pediatric, Affiliated Hospital of North Sichuan Medical College, Nanchong, China; 3 Ministry of Education Key Laboratory of Child Development and Disorders, Chongqing Key Laboratory of Pediatrics, Department of Anesthesiology, National Clinical Research Center for Child Health and Disorders, Children’s Hospital of Chongqing Medical University, Chongqing, China

**Keywords:** heart failure, myocardial fibrosis, myocardial hypertrophy, nootkatone, TGF-β1/Smad3 signaling pathway

## Abstract

Heart failure (HF) represents the clinical end stage of most cardiovascular diseases and remains a major cause of mortality, morbidity, and poor quality of life worldwide. In the present study, we use a mouse model induced by abdominal aortic constriction (AAC) that mimics HF and evaluate the potential therapeutic effects of nootkatone (NKT) on this model. Ejection fraction (EF) and fractional shortening (FS) progressively deteriorated in the AAC mice. The AAC mice were treated with NKT for 8 weeks starting on the eighth day post-AAC. Early NKT treatment prevented cardiac dysfunction in the AAC mice at 8 and 12 weeks after administration, along with thinner left ventricular posterior wall, lower left ventricular mass and ratio of heart weight/tibial length, and fewer cardiomyocyte areas. Furthermore, we found that NKT significantly reduced the expression levels of atrial natriuretic peptide (ANP) and brain natriuretic peptide (BNP), collagen types Ⅰ and Ⅲ, TGF-β1, Smad3, and the phosphorylation of Smad3. Furthermore, NKT decreased the activation of cardiac fibroblasts and myocardial fibrosis in the AAC mice. Our data suggest that NKT can delay or reverse the progression of HF after AAC and reduce myocardial hypertrophy and fibrosis possibly via inhibition of the TGF-β1/Smad3 signaling pathway.

## Introduction

Heart failure (HF) is a pathological condition that often represents the clinical end stage of various cardiovascular diseases, such as hypertension, congenital heart disease, primary or inherited cardiomyopathy, valvular disease, and coronary artery disease, and remains a major cause of mortality, morbidity, and poor quality of life worldwide (Papait et al., 2020; McDonagh et al., 2021). By 2030, the prevalence of HF in the United States is expected to reach approximately 8 million cases (Heidenreich et al., 2013). It is estimated that HF affects more than 64 million people worldwide (Savarese et al., 2023). Despite increased treatment options of HF, outcomes of HF remain suboptimal, with increasing prevalence and hospitalization rates in recent decades (Murphy et al., 2020).

Cardiac remodeling, characterized by myocardial hypertrophy and fibrosis, is a key driving force underlying the progression of HF. It is defined as a cluster of changes in the cardiac structure and function that respond to persistent physiologic or pathologic stimuli, such as myocardial damage due to various etiologies, hemodynamic changes, inflammation, or neurohormonal activation (Aimo et al., 2019). Although cardiac remodeling is compensatory in the early stages of HF development, it can deteriorate into a decompensated stage with irreversible consequences and even death without proper treatments. Cardiac remodeling is a multicellular process that consists primarily of pathological cardiac hypertrophy and fibrosis. The former is characterized by increased protein synthesis, abnormal cell growth, impaired sarcomere structure, mitochondrial dysfunction, and metabolic remodeling (Nakamura and Sadoshima, 2018), which is associated with the upregulation of atrial natriuretic peptide (ANP) and brain natriuretic peptide (BNP) expression (Sun et al., 2021). Cardiac fibrosis is a pathological response manifested by excessive deposition of extracellular matrix proteins (González et al., 2018). Myofibroblasts, transformed from fibroblasts as the main cellular source for synthesizing extracellular matrix proteins, have an augmented ability to synthesize such proteins, and their persistent presence is essential to driving fibrosis (Aujla and Kassiri, 2021). The canonical TGF-β1/Smad3 signaling pathway plays a key role in the transformation of myocardial fibrosis (Umbarkar et al., 2021).

The administration of HF has seen limited progress over the past few decades. Most drug therapies are anti-symptomatic. Therefore, there is an urgent need to develop treatment options for preventing or relieving HF in patients who are at high risks or with etiological indications of underlying HF (Rossignol et al., 2019). Nootkatone (NKT), a natural sesquiterpenoid, was first extracted from the heartwood of *Cupressus nootkatensis*. Previous studies have underscored its protective role for biological viscera, owing to its significant anti-inflammatory, antioxidant, immunomodulatory, and lipid metabolism-modulating effects (Leonhardt and Berger, 2015). Recent reports have indicated that NKT confers protective and anti-fibrotic effects in liver and renal fibrosis mouse models (Kurdi et al., 2018; Chen et al., 2021). However, it remains unclear whether NKT is effective against myocardial fibrosis and consequently for HF. Therefore, in this study, we aim to investigate the effects of early intervention of NKT in HF mice induced by pressure overload through an abdominal aortic constriction (AAC).

## Materials and methods

### Experimental animals

All C57BL/6 mice were purchased from Hunan Silaikejingda Experimental Animal Co. Ltd. (Hunan, China) and housed in controlled environments (relative humidity: 55% ± 10%; room temperature: 24 °C ± 1 °C) with a 12-h light/dark cycle (illuminated between 7:00 am and 7:00 pm). This study was approved by the Animal Research Ethics Committee of Chongqing Medical University (Chongqing, China), under the ethical approval number CHCMU-IACUC20231122010. All animals were treated according to the standard animal care guidelines established by the University Ethics Committee.

**FIGURE 1:**
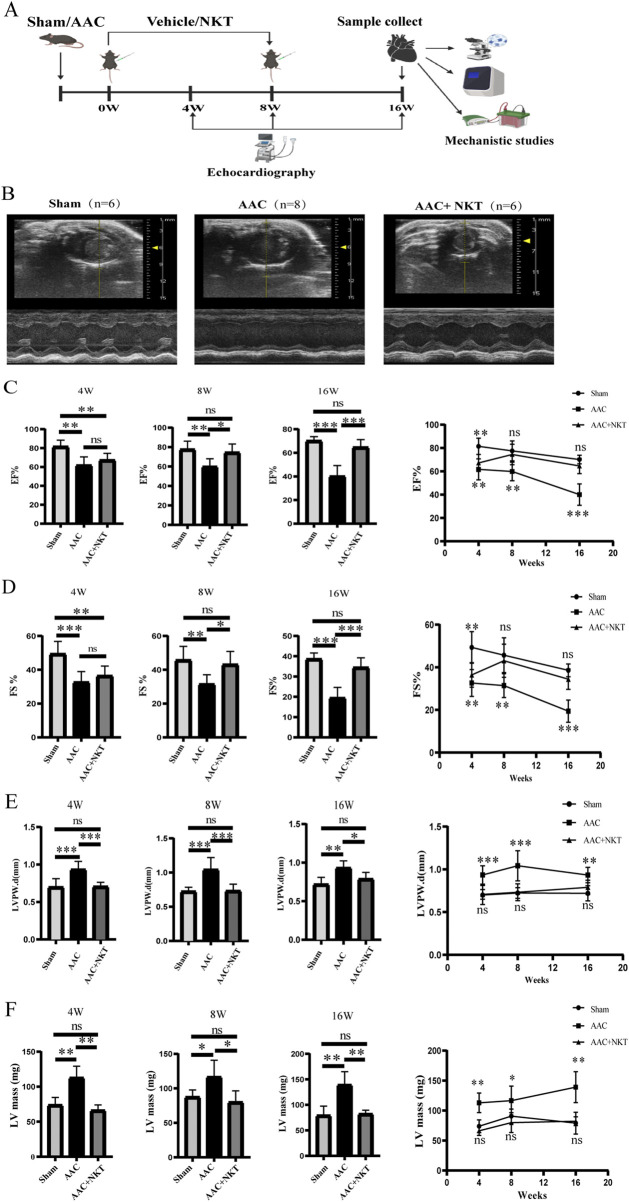
Early NKT administration prevented cardiac dysfunction induced by pressure overload at the 4th, 8th, and 16th week post-abdominal aortic constriction (AAC). **(A)** Schematic representation of this study design. **(B)** Representative M-mode echocardiographic images were obtained from short-axis views of the left ventricle (LV). **(C–F)** Quantitative measurements of cardiac functions and structures included ejection fraction (EF), fractional shortening (FS), the thickness of the posterior left ventricular wall (LVPWd), and the LV mass (n = 6–8). All values are represented as mean ± SD. *P < 0.05, **P < 0.01, and ***P < 0.001; ns, no significance.

A diagram showing the mouse treatment timeline is presented in Figure 1A. The 8-week-old male mice received AAC or sham operation as previously described (Luo et al., 2022). Abdominal aorta banding was performed with a 27-gauge needle. The needle was removed after tight banding. In contrast, mice in the sham group were subjected to the same operation without banding. The mice that underwent AAC surgery were randomly divided into the AAC group (*n* = 6) and the AAC + NKT group (*n* = 8). The sham group (*n* = 6) was considered the control. The NKT or vehicle intervention was initiated on the eighth day following AAC. NKT was dissolved in the vehicle (2% DMSO +5% Tween 80 + 40% PEG300 + 53% saline solution) according to the instructions. Mice in the AAC + NKT group were subjected to an intraperitoneal injection of 10 mg/kg NKT per day (Gairola et al., 2021) for 8 weeks. Meanwhile, mice in the AAC or sham group were administered the same amount of vehicle.

### Echocardiography

Cardiac structures and functions were assessed by echocardiography using a Vevo 3100 system (VisualSonics, North America) equipped with an MX550 probe (40 MHz) at the 4th, 8th, and 16th weeks after drug intervention. Mice were anesthetized with 1%–2% isoflurane to obtain short-axis views of the left ventricle (LV) at the level of the midpapillary muscle under M-mode. LV ejection fraction (EF), fractional shortening (FS), LV internal dimensions at end-diastole and end-systole (LVIDd and LVIDs), LV end-diastolic volume (LVEDV), and LV end-systolic volume (LVESV) were acquired to evaluate the systolic function (Deng et al., 2021). All data were analyzed using *in vivo* echocardiography report software.

### Cardiac morphology analysis

All treated mice were anesthetized and sacrificed by cervical dislocation at the 16th week after administration. The chest was opened through sternotomy to expose the heart, and the right atrium was carefully snipped, followed by perfusion with phosphate-buffered saline (PBS) from the apex until the remaining heart tissues turned pale. Then the heart was removed, rinsed with PBS, dried on absorbent paper, weighed, and photographed under natural light. Body weight (BW), heart weight (HW), and tibial length (TL) measurements and the calculated ratios of HW/TL and BW/TL were obtained to assess cardiac morphological changes after pressure overload (Saleem et al., 2018).

### Histopathological staining

Paraffin-embedded heart tissue was obtained as previously described (Zhao et al., 2021), sliced into 5-µm-thick sections on a microtome, and then subjected to hematoxylin and eosin (HE) staining and Masson’s trichrome staining. The sections were observed under light microscopy (Olympus) by an experienced pathologist in a blinded manner. ImageJ software was used for analysis of the section images.

Cell size was analyzed using fluorescein isothiocyanate-labeled wheat germ agglutinin stain (WGA, 1:500, Sigma, USA) (Wang et al., 2019). After deparaffinization, the sections were placed in a container filled with EDTA antigen repair buffer (pH 8.0) and boiled at medium power for 8 min and then at low power for 7 min in a microwave oven. After the addition of the WGA working solution, the container was incubated at room temperature for an additional 30 min. All sections with the nuclei stained with DAPI were sealed, examined, and photographed under a fluorescence microscope. ImageJ software was used for analysis of the section images.

### Immunofluorescence staining

After fixation with 4% paraformaldehyde for 24 h and subsequent dehydration in a gradient of 10%, 20%, and 30% sucrose, the heart tissues were embedded with optimal cutting temperature compound and then sectioned into 8-µm cryosections using an ultra-cryotome. Sections sealed with 5% bovine serum albumin and PBS were incubated with the anti-Col I monoclonal antibody (1:200, ServiceBio, China) and the anti-Col III monoclonal antibody (1:200, ServiceBio, China) overnight. Subsequently, the sections were incubated with the Alexa Fluor 488 goat anti-rabbit fluorescent secondary antibody (1:400, ServiceBio, China). Cell nuclei were stained with DAPI. Sections were visualized using a laser confocal microscope.

### Western blotting

The frozen heart tissues were thawed and homogenized in the ice-cold RIPA buffer containing a protease inhibitor and a phosphatase inhibitor (100:10:10). The protein concentrations were measured using an ultra-micro protein analyzer with 2 μL of the supernatant sample. Total protein lysates (20 μg) of each group were resolved by gel electrophoresis on 7.5% SDS-polyacrylamide gels, transferred to a nitrocellulose membrane, and blocked with protein-free rapid blocking buffer (1×) at room temperature for 10 min. The membrane was incubated with primary antibodies against collagen type I (Col I, 1:500, ServiceBio, China), collagen type III (Col III, 1:500, ServiceBio, China), HSP-90 (1:2000, OriGene, China), phosphorylated Smad3 (p-Smad3, 1:4000, Wanleibio, China), Smad3 (1:4000, Proteintech, China), transforming growth factor (TGF-β1, 1:5000, Proteintech, China), and β-actin (1:2000, EPITMICS, China), followed by incubation with corresponding HRP-labeled secondary antibodies (ServiceBio, China). Immunoblots were visualized with the ECL kit using the imaging system (Bio-Rad, USA), and quantitative analysis was performed using Image Lab software (National Institute of Mental Health, USA).

### Quantitative RT-PCR

Total RNA from mouse heart tissues was extracted using TRIzol reagent (Takara, China), and reverse transcription was performed using the PrimeScript™ RT-PCR kit (Takara, China). Relative expression levels of target genes were detected using SYBR Green Master Mix (Q121-02; Vazyme). The sequences of primers used for quantitative RT-PCR are shown in Table 1. The data were processed using the 2^−ΔΔCt^ method (Livak). The 2^−ΔΔCt^ values were recorded and entered into GraphPad Prism software for statistical analysis and graphing.

**TABLE 1:** Sequences of primers used for quantitative RT-PCR.

Gene	Forward	Reverse
*ANP*	TGG​GCT​TCT​TCC​TCG​TCT​T	CTT​CTC​CTC​CAG​GTG​GTC​TA
*BNP*	ATC​TCC​TGA​AGG​TGC​TGT​CC	ATC​CGG​TCT​ATC​TTG​TGC​C
*Col I*	GCT​TCC​TGC​CTC​AGC​CAC​CT	AGC​CCT​CGC​TTC​CGT​ACT​CG
*Col III*	GCC​CAC​AGC​CTT​CTA​CAC​CT	ACC​CAT​TCC​TCC​CAC​TCC​A
*GAPDH*	TGC​CCA​GAA​CAT​CAT​CCC​T	GGT​CCT​CAG​TGT​AGC​CCA​AG

### Statistical analysis

All data are presented as mean ± standard deviation and were analyzed using one-way ANOVA followed by post-hoc tests using the least significant difference (LSD) method. Differences were statistically significant when a *P*-value was less than 0.05. All statistical analyses were conducted using GraphPad Prism (version 8.3.0).

## Results

### Early intervention with NKT prevented the progression of pressure overload-induced HF

LV systolic function, as reflected by a significant reduction in EF and FS, showed progressive deterioration in AAC mice compared to that in the sham mice after surgery (*P* < 0.05, Figures 1B–D). Four weeks after treatment, EF and FS of the LV in the AAC mice were 24.27% and 33.83% lower than those in the sham mice, and the LV mass was 52.86% higher. In addition, the end-diastolic LV posterior wall (LVPWd) thicknesses were also elevated by 32.85%, suggesting a heart enlargement, ventricular remodeling, myocardial hypertrophy, and HF caused by pressure overload. These differences were more significant at the 16th week post-treatment (Figures 1C–F; Table 2). At 16 weeks, the LV mass of the AAC mice was 75.63% higher than that of the sham mice, along with a 42.77% decrease in EF and a 49.69% reduction in FS. Furthermore, the significantly enlarged hearts were found in the AAC mice, as manifested by increased LVIDd, LVIDs, LVEDV, and LVESV.

**TABLE 2:** Echocardiography after treatment.

Parameter	4 weeks			8 weeks			16 weeks		
	Sham (n = 6)	AAC (n = 6)	AAC + NKT (n = 8)	Sham (n = 6)	AAC (n = 6)	AAC + NKT (n = 8)	Sham (n = 6)	AAC (n = 6)	AAC + NKT (n = 8)
HR (beats/min)	477.5 ± 11.74	473.17 ± 7.88	477.09 ± 12.7	479.5 ± 7.02	475.5 ± 10.75	485 ± 6.18	481.83 ± 8.29	484.33 ± 9.36	478.5 ± 7.91
EF%	81.43 ± 6.39**	61.67 ± 8.24	67.19 ± 6.81	77.52 ± 7.9**	59.91 ± 7.32	74.48 ± 8.18^#^	70.12 ± 3.34**	40.01 ± 8.4	64.7 ± 6.17^##^
FS%	49.33 ± 6.78**	32.64 ± 5.76	36.4 ± 5.39	45.65 ± 7.44*	31.41 ± 5.11	43.05 ± 7.25^#^	38.6 ± 2.76**	19.42 ± 4.76	34.43 ± 4.5^##^
LVIDd (mm)	3 ± 0.2	3.45 ± 0.25	3.12 ± 0.18	3.19 ± 0.25	3.57 ± 0.36	3.46 ± 0.25	3.26 ± 0.26*	4.18 ± 0.4	3.16 ± 0.24^#^
LVIDs (mm)	1.53 ± 0.27*	2.34 ± 0.35	1.98 ± 0.21	1.74 ± 0.3	2.46 ± 0.37	1.99 ± 0.36	2 ± 0.19*	3.37 ± 0.4	2.07 ± 0.24^#^
LVPWd (mm)	0.7 ± 0.1*	0.93 ± 0.1	0.71 ± 0.05^#^	0.72 ± 0.06**	1.04 ± 0.16	0.73 ± 0.09^##^	0.72 ± 0.08**	0.93 ± 0.08	0.79 ± 0.08^##^
LVEDV (ul)	35.26 ± 5.82	49.55 ± 8.58	38.68 ± 5.52	40.93 ± 7.58	54.31 ± 13.74	50.06 ± 8.26	43.36 ± 8.54**	78.68 ± 16.7	40.06 ± 7.3^##^
LVESV (ul)	6.72 ± 3.06	19.59 ± 7.12	12.71 ± 3.3	9.36 ± 4.13	22.22 ± 8.9	13.26 ± 5.65	13.02 ± 3.15**	47.49 ± 12.75	14.3 ± 4.27^##^
LV mass (mg)	73.84 ± 9.87**	112.87 ± 14.98	66.2 ± 7.26^##^	90.79 ± 10.66*	141.52 ± 69.54	79.95 ± 15.5^#^	79.16 ± 16.6**	139.03 ± 23.61	82.18 ± 6.9^##^

Values are expressed as mean ± standard deviation (SD). HR, heart rate; EF, left ventricular ejection fraction; FS, fractional shortening; LVIDd, left ventricular internal diameter at end-diastole; LVIDs, left ventricular internal diameter at end-systole; LVPWd, left ventricular posterior wall thickness at diastole; LVEDV, left ventricular end-diastolic volume; LVESV, left ventricular end-systolic volume; LV mass, left ventricular mass. * represents *P* < 0.05, and ** represents *P* < 0.01, sham group versus AAC group.

# represents *P* < 0.05, and ## represents *P* < 0.01, AAC + NKT group versus AAC group.

Intriguingly, the AAC mice treated with NKT exhibited an increase in EF and FS at the 8th week, which continued through the 16th week after NKT administration, compared to that of the AAC mice without NKT treatment, implying that cardiac dysfunction was prevented by NKT. Meanwhile, LVPWd remained remarkably stable in the AAC mice treated with NKT compared to the AAC mice without NKT at the 4th, 8th, and 16th weeks after treatment (*P* < 0.05, Figure 1E; Table 2). Additionally, the pronounced changes in LVIDd, LVIDs, LVEDV, and LVESV in the AAC mice were maintained at a sham level with NKT treatment at the 16th week, along with a lower level of the LV mass, compared to the AAC mice (*P* < 0.05, Table 2). Early treatment with NKT effectively prevented cardiac dysfunction as assessed by echocardiography.

### Early treatment of NKT reduced pressure overload-induced cardiac hypertrophy

**FIGURE 2:**
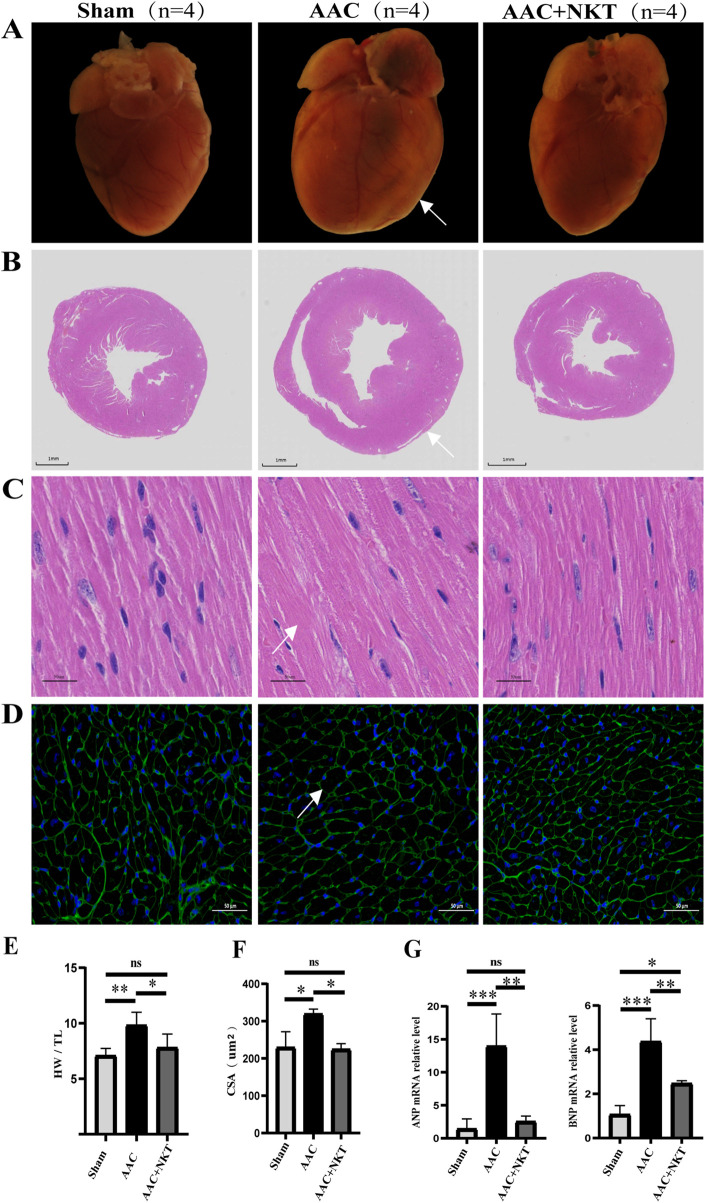
Early NKT administration preserved cardiac hypertrophy induced by pressure overload. **(A)** Myocardial hypertrophy was assessed using whole-heart photographs (n = 4). **(B)** H&E staining of heart tissue in short-axis view; scale bar, 1 mm (n = 4). **(C)** Detailed view of H&E-stained tissue; scale bar, 50 μm m (n = 4). **(D)** WGA staining; scale bar, 50 μm (n = 4). **(E)** Ratio of heart weight (HW)/tibial length (TL) (n = 4). **(F)** Quantitative measurements of the cross-sectional area (CSA) of the LV cardiomyocytes (n = 4). **(G)** mRNA expression level of ANP and BNP in heart tissue (n = 6–8). *P < 0.05, **P < 0.01, and ***P < 0.001; ns, no significance.

Sixteen weeks after treatment, the HW/TL ratio in the AAC mice increased by 38.65% compared with that in the sham mice (*P* < 0.01, Figure 2E; Table 3). However, NKT administration significantly prevented pathological heart enlargement and cardiac hypertrophy in the AAC mice (*P* < 0.01, Figures 2A–F). In addition, the HW/TL ratio was significantly reduced by 20.45% at week 16 (*P* < 0.05, Figure 2E; Table 3). The average cross-sectional area of the LV cardiomyocytes assessed via WGA staining was used to calculate cardiomyocyte size, demonstrating that the area was 44.0% larger in the AAC mice than that in the sham mice (*P* < 0.05, Figure 2F). In contrast, a 31.68% reduction in the mean cardiomyocyte cross-sectional area was observed in the AAC mice with NKT supplementation (*P* < 0.05, Figure 2F). A remarkable upregulation of transcription for the myocardial hypertrophy marker genes such as *ANP* and *BNP* was detected in the AAC mice compared to that in the sham mice (*P* < 0.005, Figure 2G), whereas the mRNA levels of these markers were significantly decreased in the AAC + NKT group (*P* < 0.01, Figure 2G).

**TABLE 3:** Cardiac morphology at 16 weeks after treatment.

Parameter	Sham	AAC	AAC + NKT
BW (g)	28.42 ± 0.79	30.37 ± 1.98	28.63 ± 1.34
HW (mg)	126.17 ± 10.29***	184.33 ± 21.70	147.50 ± 22.64^#^
TL (mm)	17.79 ± 0.13	18.74 ± 0.27	18.84 ± 0.58
BW/TL	1.60 ± 0.04	1.62 ± 0.09	1.52 ± 0.06
HW/TL	7.09 ± 0.58**	9.83 ± 1.06	7.82 ± 1.11^#^

Values are expressed as mean ± standard deviation (n = 6 mice per group); BW, body weight; HW; heart weight; TL, tibial length. ** represents *P* < 0.01, and *** represents *P* < 0.001, sham group versus AAC group. # represents *P* < 0.05, AAC + NKT group versus AAC group.

### Early treatment with NKT inhibited pressure overload-induced myocardial fibrosis

**FIGURE 3:**
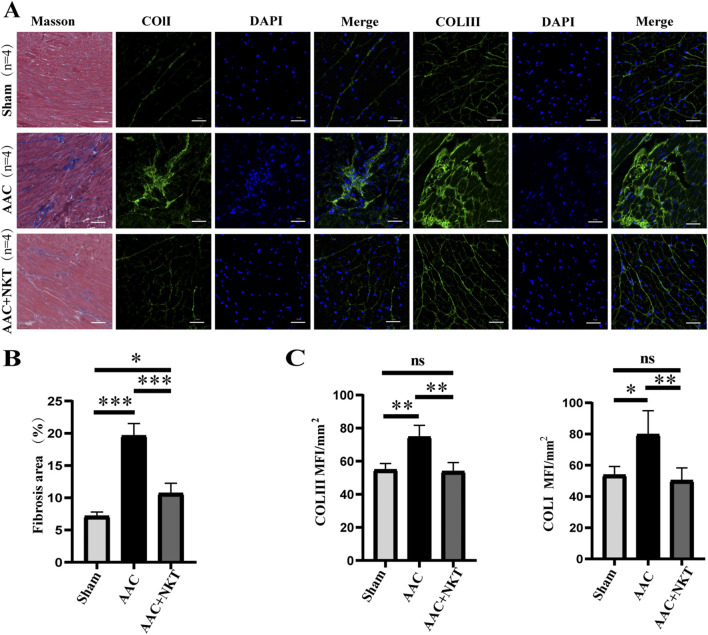
Early NKT administration inhibited cardiac fibrosis induced by pressure overload (n = 4). **(A)** Representative histology of cardiac sections stained with Masson’s trichrome staining and immunofluorescence staining for Col Ⅰ, Col Ⅲ, and DAPI of the left ventricle; scale bar, 50 μm. **(B)** Fibrotic area of the heart section. **(C)** Mean fluorescence intensity of Col Ⅰ and Col Ⅲ. *P < 0.05, **P < 0.01, and ***P < 0.001; ns, no significance.

**FIGURE 4:**
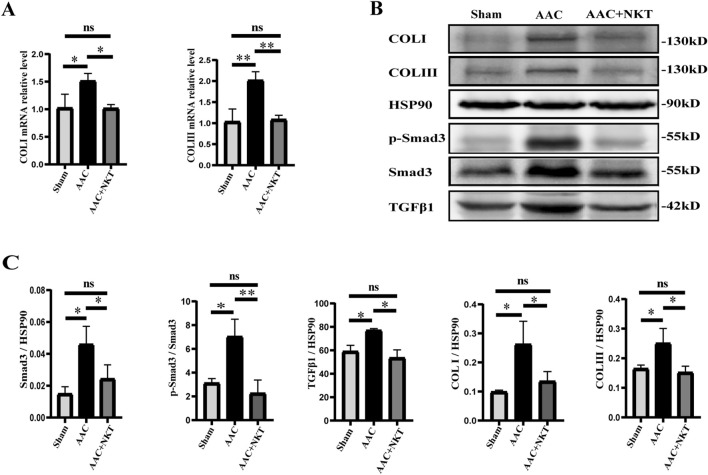
Early NKT administration downregulated the TGF-β1/Smad3 signaling pathway (n = 6–8). **(A)** mRNA expression of Col Ⅰ and Col Ⅲ in heart tissue. **(B)** Protein expression of Col Ⅰ, Col Ⅲ, HSP-90, TGF-β1, Smad3, and p-Smad3 in heart tissue. **(C)** Protein quantitative analysis of Col Ⅰ, Col Ⅲ, HSP-90, TGF-β1, Smad3, and p-Smad3. *P < 0.05, **P < 0.01, and ***P < 0.001; ns, no significance.

Myocardial fibrosis is an evidence of ventricular remodeling, which is associated with cardiac dysfunction (Goh et al., 2019). Myocardial tissue sections examined with Masson’s staining and immunofluorescence staining were used to determine the degree of myocardial fibrosis and demonstrate the protective effects of NKT on AAC mice. The fibrotic area in the LV myocardium was significantly increased by 1.75 folds in the AAC mice compared to that in the sham group (*P* < 0.001, Figures 3A,B); however, this elevation was markedly reduced at the 16th week following NKT treatment. Cardiac fibroblast activation was accompanied by a significant increase in mean fluorescence intensity of Col Ⅰ by 48.5% and Col Ⅲ by 36.1%; however, this augmentation was significantly inhibited by NKT treatment (*P* < 0.01, Figures 3A,C). In addition, significant upregulation of transcripts and proteins for Col Ⅰ and Col Ⅲ was observed in the AAC mice, along with inhibiting effects of NKT (*P* < 0.01, Figures 4A,B). Furthermore, compared to those in the sham mice, higher TGF-β1 and Smad3 protein expression and p-Smad3/Smad3 ratio in the myocardium were demonstrated in the AAC group (*P* < 0.01, *P* < 0.05, Figures 4B,C); however, these proteins were normalized in the AAC mice treated with NKT similar to the levels observed in the sham mice (Figures 4B,C). These results suggested that early treatment with NKT could inhibit pressure overload-induced myocardial fibrosis possibly via downregulation of the TGF-β1/Smad3 signaling pathway.

## Discussion

HF has become a serious health concern worldwide. Several epidemiological studies have consistently demonstrated that HF remains a major cause of mortality, morbidity, and poor quality of life and affects at least 26 million people worldwide (Hao et al., 2019). A survey involving 22,158 participants revealed that there was an increase in the prevalence of HF and cardiac dysfunction in the recent years (Hao et al., 2019). In the present study, we use an AAC model that was constructed to mimic the pathogenic process of HF and ventricular remodeling. Early treatment with NKT prevented pressure overload-induced cardiac dysfunction in the AAC mice. Additionally, the early treatment with NKT in AAC mice for 8 weeks could ameliorate pressure overload-induced myocardial fibrosis and hypertrophy. Furthermore, NKT attenuated cardiac fibrosis by inhibiting cardiac fibroblast activation possibly via downregulation of the TGF-β1/Smad3 signaling pathway and a reduction in collagen fiber deposition in the heart.

HF is a complex clinical syndrome characterized by impaired ventricular pumping that fails to meet the body’s metabolic demands and currently has no effective therapies (Mishra and Kass, 2021; Ge et al., 2019). Previous studies have confirmed that AAC surgery is recognized as a useful tool to induce a pressure overload-induced HF-like state and ventricular remodeling in mice, which initially leads to a compensatory hypertrophy of the heart and, subsequently, to pathological myocardial hypertrophy and fibrosis (Mou et al., 2022; Hou et al., 2021). This process simulates the clinical process of HF caused by an enhanced overload, such as aortic coarctation or hypertension, and could serve as a suitable disease model for exploring therapeutic options for HF. In this study, a pressure overload-induced AAC model was used to elucidate the different stages of HF and to compare the protective effects of NKT in mice with HF. Four weeks after treatment (5 weeks after AAC surgery), cardiac dysfunction was obvious, which was indicated by a significant reduction in EF and FS, and a pronounced increase in LVPW and LV mass was observed in the AAC mice. Over time, the response to chronic pressure overload progressed to a further deteriorative state, leading to limited ejection capacity and ventricular dilatation, suggesting progressive HF. One week after AAC surgery, NKT, a natural sesquiterpenoid, was administered to treat the mice with AAC for 8 weeks. The structural and functional changes in HF were prevented in the AAC mice, and this effect persisted for up to 16 weeks or longer.

Ventricular remodeling including myocardial hypertrophy and fibrosis is the result of the changes in cardiomyocytes, cardiac fibroblasts, and other cells, which plays a key role in HF development (Cohn et al., 2000). Multiple chemical and physical factors, including angiotensin and circulating pressure overload, could stimulate hypertrophy of ventricular cardiomyocytes. Furthermore, the development of HF is associated with increased levels of ANP and BNP (Mou et al., 2022). TGF-β1, a member of the TGF-β superfamily involved in the regulation of cell growth and differentiation, is a strong inducer of cardiac hypertrophy (Fujiu and Nagai, 2014). Several studies have indicated that TGF-β1 is produced by cardiac fibroblasts and TGF-β1 can induce cardiomyocytes to secrete the connective tissue growth factor (CTGF) (Fujiu and Nagai, 2014; Lv et al., 2021). Myocardial hypertrophy induced by pressure overload can be mediated by CTGF from cardiomyocytes via the TGF-β1 signaling pathway. In the present study, we observed that the NKT-treated mice after AAC revealed a smaller cardiac size and cardiomyocyte cross-sectional area with a lower activation level of the TGF-β1/Smad3 signaling pathway, suggesting that NKT might inhibit cardiac hypertrophy and prevent HF by downregulating the TGF-β1/Smad3 signaling pathway in cardiomyocytes.

Cardiac fibrosis, a common feature of pressure overload or myocardial injury, is caused by an imbalance between the production and degradation of Col I and Col III in the extracellular matrix (ECM), thereby inducing ventricular remodeling and impairing cardiac functions (Xu et al., 2021). TGF-β is also the major factor in cardiac fibrosis following pressure overload (Pardali et al., 2017; Yao et al., 2020), and Smad3 and active p-Smad3, the downstream regulators, are the target proteins for TGF-β signal transduction and mediate the transport of TGF-β signals from cell membrane receptors to the nucleus, sequentially upregulating the transcription of multiple genes that promote tissue fibrosis (Wang et al., 2020; Flanders, 2004). Cardiac fibroblasts activate and proliferate and differentiate into myofibroblasts, which are key contributors to cardiac fibrosis (Gibb et al., 2020). Therefore, inhibition of the key pro-fibrotic stimuli is considered a developing strategy against ventricular remodeling and HF. In our study, a mouse model of pressure overload-induced HF was constructed using AAC surgery. The TGF-β1 signaling pathway was significantly downregulated, and the phosphorylation of Smad3 was almost completely blocked after NKT treatment, along with the decreased expression of ECM structural proteins Col I and Col III, indicating that suppression of the TGF-β1/Smad3 signaling pathway likely contributes to the protective effects of NKT.

NKT, a sesquiterpene derivative, is mainly isolated from grapefruit. Some studies have reported that it possesses a wide range of pharmacological effects, such as anti-cancer, anti-inflammatory, anti-bacterial, antihyperlipidemic, antiplatelet, neuroprotective, renoprotective, and hepatoprotective effects (Dai et al., 2023; Bezerra Rodrigues Dantas et al., 2020; Yong et al., 2022; Yoo et al., 2020; Meeran et al., 2020; Seo et al., 2011). NKT treatment could mitigate carbon tetrachloride (CCl_4_)-induced hepatic changes including elevated serum aspartate and alanine transaminase levels, hepatic oxidative and nitrosative stress, increased cell apoptosis, and enhanced expression of pro-inflammatory cytokines. These effects contribute to hepatoprotective and antifibrotic outcomes (Kurdi et al., 2018). In addition, NKT can reduce the accumulation of lipids in the myocardium induced by the subcutaneous injection of isoproterenol by inhibiting HMG-CoA-R activity and promoting high-density lipoprotein (HDL) cholesterol metabolism. These effects suggest that NKT could protect the heart and correct lipid abnormalities and dyslipidemia (Meeran et al., 2020). A previous study investigated the antiplatelet effects of the *Cyperus rotundus* EtOH extract (CRE) and its constituent compounds (Seo et al., 2011). Of its eight components, NKT was found to possess the most potent inhibitory effect on collagen-, thrombin-, and arachidonic acid-induced platelet aggregation. Meanwhile, NKT-treated mice exhibited significantly prolonged bleeding times, suggesting that NKT might have therapeutic potential in preventing platelet-associated cardiovascular diseases. However, there is limited exploration regarding its cardioprotective activities, and only few reports show that NKT could improve myocardial injury induced by doxorubicin or myocardial infarction by reducing oxidative stress (Meeran et al., 2020; Al-Salam et al., 2022). The data from this study elucidated, for the first time, the cardioprotective effects of NKT against pressure overload-induced HF and ventricular remodeling, along with its key pathological pathways.

In summary, our study demonstrates that early treatment with NKT can ameliorate pressure overload-induced cardiac remodeling and improve cardiac function in mice with HF caused by AAC. Our data also suggest that downregulation of the TGF-β1/Smad3 signaling pathway is likely the key mechanism underlying the protective effects of NKT.

## Data Availability

The original contributions presented in the study are included in the article/Supplementary Material; further inquiries can be directed to the corresponding author.
